# Review of "The Cell Cycle: Principles of Control" by David O. Morgan

**DOI:** 10.1186/1747-1028-2-27

**Published:** 2007-09-17

**Authors:** Mignon A Keaton

**Affiliations:** 1 Department of Pharmacology and Cancer Biology, Box 3813, Duke University Medical Center, Durham, NC 27710, USA

"The Cell Cycle: Principles of Control" by David Morgan is the second publication in the Primers In Biology series from New Science Press Ltd. This text aims to provide "a clear and concise guidebook" to our knowledge of the complex network of signaling pathways, regulatory circuits, and biochemical machines employed during cell reproduction. The result is a well-written book that is ideal for both students and seasoned scientists who are newcomers to the cell cycle field. Upper-level undergraduates and new graduate students will find it easy to follow, thanks to numerous color figures whose schematics are used throughout the book. Each of the twelve chapters is divided into modular two-page sections that address specific topics (histone synthesis in S phase, for example). This webpage-like format is ideal for students who are used to retrieving information online, but readers looking for a thorough discussion of a topic may be frustrated by the limitations that a two-page partition imposes. Although molecular details are often included, this text is not a comprehensive review of the field. Instead, each chapter provides background for further reading and includes references for reviews and primary literature in each section.

A welcome aspect of the book is Morgan's attempt to integrate information gained from the study of different model organisms. In order to do so, Chapter 2 is dedicated to the organization of (both budding and fission) yeast, *Drosophila*, *Xenopus*, and mammalian cell cycles. This is especially helpful for understanding the data figures in the book as well as those in the primary literature. While most sections compare regulatory mechanisms in multiple systems (usually comparing budding yeast with mammalian cells), some sections only discuss the system in which the most complete information is known. Tables correlating protein homologues across multiple species are present throughout the text.

Morgan introduces the "cell-cycle control system" as the regulatory network that acts as a biological timer to ensure the execution of cell cycle events in a timely and consistent way. Cyclin-dependent kinases (CDKs) are the major components of this system, and an understanding of CDK regulation is an important foundation for studying the cell cycle. Thus, CDK regulation by phosphorylation and cyclin degradation is introduced before any stage of the cell cycle is covered in detail. A main focus of Chapter 3 is the inherent bistability of the system that allows for switch-like transitions between G1/S and G2/M and the establishment of a self-perpetuating oscillator. Sections 3–7, 3–8, and 3–11 are the best explanations of these mechanisms that I've read to date.

After establishing the principles of CDK regulation, how the cell-cycle control system executes DNA replication, nuclear division, and cytokinesis is described in Chapters 4–8. This allows Morgan to address seamlessly both the underlying mechanisms and the regulation of those mechanisms at each step in the cell cycle. Ultimately, this approach gives the reader a more fluid view of the cell cycle and a firmer understanding of how regulatory circuits in one phase can mediate regulation of the following phases.

Although this book is entitled "The Cell Cycle: Principles of Control," the mechanics of cell cycle events are equally addressed. The mitotic spindle is discussed in detail in Chapter 6. A thorough introduction to microtubule structure and behavior is given. The roles of microtubule motors and kinetochores in establishing a spindle with bi-oriented chromosomes is discussed. Morgan presents the enigmatic processes of kinetochore attachment, microtubule flux, and chromosome congression by discussing proposed models in the context of published data. Similarly, the mechanics of the actin-myosin contractile ring during cytokinesis is detailed in Chapter 8 and the structural connections between homologous chromosomes during meiosis are emphasized in Chapter 9. Researchers whose work focuses on other aspects of the cell cycle may particularly appreciate the attention given to these events.

A review of the cell cycle would not be complete without a discussion of the effects that extracellular signaling pathways have on cell proliferation. Morgan goes a step further by also discussing the regulation of cell size and the influence of cell metabolism on the cell-cycle control system. In addition, the negative effects of hyperproliferation and telomere shortening are included.

One of the attractive aspects of this book is Morgan's excellent description of the principles of tumorigenesis in Chapter 12. The development of improved detection methods and treatments for cancer is one of the main goals of understanding cell cycle regulation. Researchers will appreciate the sections covering the different mechanisms of acquiring mutations and genomic instability, the nomenclature describing the tissue specificity of tumors, and the genes most commonly mutated in specific forms of cancer. This chapter is a very good primer for reading cancer-related studies, which can often be very clinical in nature.

Overall, this book offers an excellent portrait of the fascinating complexity of cell division. The principles behind the detailed mechanisms of ensuring timely and controlled progression through the cell cycle are well communicated. Morgan often cross-references related sections, allowing the reader to skip around chapters as needed. Published data is presented in a clear and useful way. For these reasons, I believe this book would be an ideal text for an advanced undergraduate or graduate-level class. As a young researcher in the cell cycle field, I also believe this book has the potential to inspire students to join other researchers in the endeavor to further our understanding of cell reproduction.

